# Effects of Anesthetic Technique on the Occurrence of Acute Kidney Injury after Spine Surgery: A Retrospective Cohort Study

**DOI:** 10.3390/jcm10235653

**Published:** 2021-11-30

**Authors:** Jiwon Han, Ah-Young Oh, Chang-Hoon Koo, Yu Kyung Bae, Yong-Tae Jeon

**Affiliations:** 1 Department of Anesthesiology and Pain Medicine, Seoul National University Bundang Hospital, Gumi-ro, Bundang-gu, Seongnam-si 13620, Korea; yesuroon@gmail.com (J.H.); ohahyoung@hanmail.net (A.-Y.O.); vollock9@gmail.com (C.-H.K.); vansuri@naver.com (Y.K.B.); 2 Department of Anesthesiology and Pain Medicine, Seoul National University College of Medicine, Daehak-ro, Jongno-gu, Seoul 03080, Korea

**Keywords:** acute kidney injury, spine surgery, total intravenous anesthesia, volatile anesthetic agent

## Abstract

The effects of anesthetics on acute kidney injury (AKI) after spine surgery have not been evaluated fully. This study compared propofol-based total intravenous anesthesia (TIVA) and volatile anesthetics in the development of AKI after spine surgery. This retrospective study reviewed patients who underwent spine surgery between 2015 and 2019. A logistic regression analysis was performed to identify risk factors for AKI. Additionally, after propensity score matching, the incidence of AKI was compared between TIVA and volatile groups. Of the 4473 patients, 709 were excluded and 3764 were included in the logistic regression. After propensity score matching, 766 patients from each group were compared, and we found that the incidence of AKI was significantly lower in the TIVA group (1% vs. 4.2%, *p* < 0.001). In the multivariate logistic regression analysis, the risk factors for postoperative AKI were male sex (OR 1.85, 95% CI 1.18–3.06), hypertension (OR 2.48, 95% CI 1.56–3.94), anemia (OR 2.66, 95% CI 1.76–4.04), and volatile anesthetics (OR 4.69, 95% CI 2.24–9.84). Compared with volatile anesthetics, TIVA is associated with a reduced risk of AKI for patients who have undergone spine surgery.

## 1. Introduction

Postoperative acute kidney injury (AKI) is associated with mortality and quality of recovery. It is not just an indicator of multiorgan dysfunction but is itself an important independent factor for postoperative outcome [1].

Propofol protects the vital organs, including the kidneys [2]. Animal experiments found that propofol has anti-inflammatory, anti-oxidative, anti-apoptotic, and immunomodulatory effects, and it reduces ischemia/reperfusion injury in kidney cells [3,4,5]. However, it is not yet clear whether propofol has clinically meaningful renoprotective effects compared with volatile anesthetics.

Spine surgery has several risk factors for the development of AKI, such as increased intra-abdominal pressure due to being in a prone position, hemodynamic changes, surgical inflammation, embolic events, use of intraoperative vasoactive drugs, blood loss, and hemodilution [6,7]. However, few studies have investigated AKI after spine surgery, especially anesthesia-related risk factors. Therefore, this study investigated the association between the incidence of postoperative AKI and the main anesthetic agents used in spine surgery. The secondary purpose was to investigate the risk factors for AKI after spine surgery.

## 2. Materials and Methods

### 2.1. Study Design and Patient Data

This retrospective observational study adhered to the principles of the Declaration of Helsinki and was approved by the Institutional Review Board of Seoul National University Bundang Hospital (approval number: B-2006/616-120; approval date: 16 June 2020). The requirement for informed consent was waived due to the retrospective nature of the study. This manuscript complied with the STROBE guidelines. The data were based on the electronic medical records of patients 20 years or older who underwent spine surgery between 1 January 2015 and 31 December 2019 at Seoul National University Bundang Hospital. The dataset was limited to a period of 5 years to limit confounding changes in surgical and anesthesia practices. Patients who underwent emergency surgery, metastatic tumor removal, or surgeries with local anesthesia were excluded from the analysis. Thoracolumbar surgery was also excluded because total intravenous anesthesia (TIVA) was used in all cases for neurophysiological monitoring.

The following data were obtained, which may represent potential risk factors for postoperative AKI. Demographics and preoperative medical history data included hypertension, diabetes mellitus, coronary artery disease, dyslipidemia, smoking, anemia, laboratory values of glomerular filtration rate (GFR), serum creatinine, and medications such as non-steroidal anti-inflammatory drugs (NSAIDs), angiotensin-converting enzyme inhibitors (ACEi), angiotensin receptor blockers (ARB), and diuretics. Surgical data included the surgical range of spine level and operation time. Anesthetic data included intraoperative hypotension (defined as a mean arterial pressure below 55 mmHg), intraoperative hypothermia (defined as a body temperature measured at the esophagus below 35 °C), the amount of transfusion, crystalloid, colloid, estimated blood loss, and the need for intraoperative inotropes or vasopressors. In addition, the main general anesthetic agent was recorded. Propofol and remifentanil continuous infusions using target-controlled infusion (Fresenius Vial, Brezins, France) were used for TIVA. For volatile anesthetics, sevoflurane or desflurane with remifentanil was used for balanced anesthesia. Serum creatinine levels before surgery and up to 7 days postoperatively were collected.

### 2.2. Definition of AKI

The primary endpoint was the occurrence of postoperative AKI. Kidney Disease: Improving Global Outcomes (KDIGO) criteria were used to define and grade AKI [8]. In our institution, serum creatinine is measured 1 month prior to scheduled surgery; this value was defined as the baseline serum creatinine. AKI stage 1 was defined as a 0.3 mg/dL increase in serum creatinine or a 1.5–1.9 fold increase over the preoperative level. AKI stage 2 was defined as a 2.0–2.9 fold increase in serum creatinine relative to the preoperative level. AKI stage 3 was defined as a 4.0 mg/dL increase in serum creatinine, a more than three-fold increase over the preoperative level, or the initiation of renal replace therapy [8].

### 2.3. Statistics

The main anesthetic agents were not randomly assigned; therefore, the propensity score matching (PSM) method was used to balance the covariates between the TIVA and volatile groups. The following variables were matched: age, sex, body mass index (BMI), comorbidities such as hypertension, diabetes mellitus, coronary artery disease, dyslipidemia, smoking, and anemia, the use of NSAIDs, ACEi, ARB, and diuretics, intraoperative hypotension, hypothermia, the intraoperative transfusion amount, crystalloid, colloid, estimated blood loss, and administration of ephedrine, phenylephrine, or norepinephrine. PSM was performed at a 1:1 ratio with the optimal method using the MatchIt package in R 3.6.1 (R Project for Statistical Computing, Vienna, Austria) [9]. Patient characteristics before and after matching were compared, and the absolute standardized mean difference (ASD) <0.2 was used to confirm the balance. Continuous variables are expressed as the mean with standard deviation and were compared using independent *t*-tests. Categorical variables are presented as numbers with percentages and were compared using the Chi-square test or Fisher’s exact test.

We conducted univariate logistic regression analysis to identify potential risk factors for AKI after spine surgery. The covariates significant at *p* < 0.1 in the univariate logistic regression analysis were included in the multivariate logistic regression analysis. The goodness of fit for the final multivariate logistic model was tested using the Hosmer and Lemeshow tests. All data analyses were performed with R 3.6.1. Statistical significance was deemed as a *p*-value less than 0.05.

## 3. Results

**Figure 1:**
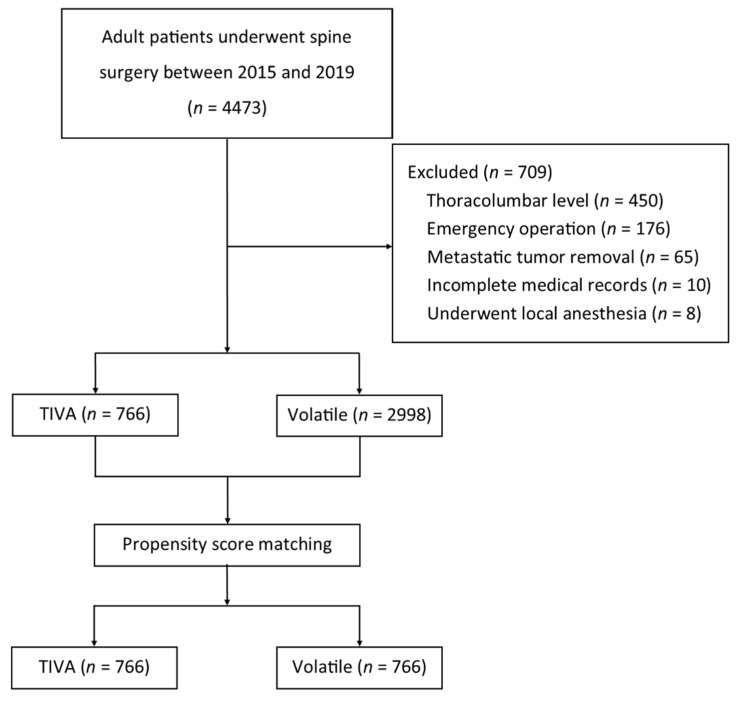
Flow diagram.

In the 5-year period from 1 January 2015 to 31 December 2019, 4473 patients over the age of 20 underwent spine surgery at Seoul National University Bundang Hospital. Overall, 709 patients were excluded for the following reasons: 450 patients who underwent thoracolumbar surgery, 176 patients who underwent emergency surgery, 65 patients who underwent surgery to remove metastatic tumors, 10 patients with incomplete medical records, and 8 patients who underwent surgeries with local anesthesia. As a result, 3764 patients who underwent spine surgery were included in the logistic regression analysis. Among these 3764 patients, 766 patients were treated with TIVA, and 2998 patients were treated with volatile anesthetic agents. After PSM, 766 patients remained in each group (Figure 1).

Table 1 compares the baseline characteristics and intraoperative parameters before and after PSM between the TIVA and volatile groups. The matched cohort ASDs were all less than 0.2. Table 2 shows that the TIVA group had a lower incidence of postoperative AKI compared with the volatile group in the matched cohort.

Table 3 summarizes the risk factors for AKI based on univariate and multivariate logistic regression analysis. After the univariate analysis, the following variables were included in the multivariate logistic regression analysis: male sex, higher American Society of Anesthesiologists (ASA) physical status, preoperative medical history of hypertension, diabetes mellitus, smoking, and anemia, medication with ACEi or ARB, and diuretics, longer surgical level, longer surgery duration (>3 h), intraoperative hypotension (MBP < 55 mmHg), and use of volatile anesthetic agents rather than TIVA. The independent risk factors for AKI after spine surgery were male sex, preoperative medical history of hypertension, anemia, and use of a volatile anesthetic agent.

Of the 3764 patients, 126 (3.3%) developed AKI as defined by the KIDGO criteria. Of these 126 patients, 125 were classified as stage 1, while only one patient was classified as stage 2. No patient developed stage 3 or required renal replacement therapy.

## 4. Discussion

This retrospective study showed that the use of TIVA with propofol is associated with a lower incidence of AKI after spine surgery compared with the use of volatile anesthetics. In addition, male sex, hypertension, and anemia were risk factors for developing AKI.

Several studies have compared the incidence of postoperative AKI between propofol and volatile anesthetic agents. In accordance with our results, some retrospective studies of nephrectomy, colorectal surgery, and major abdominal surgery demonstrated that propofol was associated with a reduced incidence of postoperative AKI [10,11,12,13]. In addition, patients treated with propofol had lower kidney specific biomarkers, pro-inflammatory cytokines, and incidences of postoperative AKI compared with those treated with sevoflurane during major abdominal or cardiac surgery [14,15]. In comparison, in lung surgery, propofol did not have superior renoprotective effects [16]. This discrepancy may be caused by a wide variety of surgery types and severity.

Our multivariate analysis revealed that male sex, hypertension, preoperative anemia, and volatile anesthetic agents were independent risk factors for developing AKI after spine surgery. Previous studies have shown that testosterone makes the kidneys vulnerable to ischemia/reperfusion injury, and estrogen has renoprotective effects as an antioxidant [17]. High blood pressure makes nephrons narrow, weak, or hard, and anemia reduces the delivery of oxygen to the kidneys. Therefore, preoperative hypertension and anemia predispose the kidneys to ischemic insult [18,19].

Of the patients who developed AKI, only one was stage 2; the others were stage 1. Although relatively mild, stage 1 AKI is an independently risk factor for post-cardiac surgery infection, intensive care unit and hospital stays, and early and late mortality [20,21]. Therefore, since the choice of anesthetic agent is a modifiable risk factor for AKI, the results of this study are meaningful in that the choice of anesthetic technique could help improve the outcome of surgical patients.

The AKI incidence rate of 3.3% is relatively low compared with other studies because only serum creatinine was used for diagnosing AKI and urine output was not used, owing to varying periods of urinary catheter use and insufficient records about voiding in the postoperative period. If the urine output had been applied to the definition of AKI, there may have been a higher occurrence of AKI.

This study has several limitations. The major limitation was its retrospective nature, which is vulnerable to selection bias. Although we tried to control for confounding factors by collecting comprehensive data and performing PSM, the possibility of bias remains due to the variability in surgical and postoperative management among different medical service providers. Second, the AKI incidence may have been underestimated because urine output was not included among the AKI diagnostic criteria. Third, this study examined spine surgery cases at a single institution; therefore, the results may not be generalizable to other populations. Fourth, postoperative recovery quality, such as pain scores, nausea and vomiting, complications other than AKI, and mortality, were not investigated.

## 5. Conclusions

This retrospective study suggests that the use of propofol as the main general anesthetic agent in spine surgery is associated with a reduced risk of postoperative AKI compared with the use of volatile anesthetic agents. Further randomized prospective studies are required to confirm these findings.

## Figures and Tables

**Table 1:** Comparison between TIVA (total intravenous anesthesia) group and volatile anesthesia group for baseline characteristics before and after propensity score matching (PSM).

	Before PSM			After PSM		
Variables	TIVA (*n* = 766)	Volatile (*n* = 2998)	ASD	TIVA (*n* = 766)	Volatile (*n* = 766)	ASD
Age (years)	60.6 ± 13.5	64.1 ± 13.0	0.27	60.6 ± 13.5	61.5 ± 13.7	0.06
Male sex	470 (61.4%)	1457 (48.6%)	0.26	470 (61.4%)	468 (61.1%)	0.05
BMI (kg m^−2^)	27.9 ± 82.4	27.0 ± 25.2	0.01	27.9 ± 82.4	26.1 ± 14.4	0.02
ASA						0.062
1	219 (28.6%)	811 (27.1%)		219 (28.6%)	218 (28.5%)	
2	464 (60.6%)	1848 (61.6%)	0.03	464 (60.6%)	450 (58.7%)	
3 + 4	83 (10.8%)	339 (11.3%)	0.04	83 (10.8%)	98 (12.8%)	
Comorbidities						
Hypertension	335 (43.7%)	1510 (50.4%)	0.13	335 (43.7%)	336 (43.9%)	0.003
Diabetes mellitus	18 (24.3%)	723 (24.1%)	0.003	186 (24.3%)	211 (27.5%)	0.075
Coronary disease	70 (9.1%)	304 (10.1%)	0.03	70 (9.1%)	80 (10.4%)	0.044
Dyslipidemia	52 (6.8%)	197 (6.6%)	0.01	52 (6.8%)	58 (7.6%)	0.03
Smoking	358 (46.7%)	1011 (33.7%)	0.28	358 (46.7%)	361 (47.1%)	0.008
Anemia	128 (16.7%)	561 (18.7%)	0.05	128 (16.7%)	127 (16.6%)	0.004
Medication						
NSAIDs	113 (14.8%)	451 (15%)	0.01	113 (14.8%)	121 (15.8%)	0.029
ACEi/ARB	177 (23.1%)	670 (22.4%)	0.02	177 (23.1%)	202 (26.4%)	0.076
Diuretics	60 (7.8%)	215 (7.2%)	0.03	60 (7.8%)	62 (8.1%)	0.01
Surgical parameter						
Surgical level	2.2 (1.3)	1.6 (1.0)	0.57	2.2 (1.3)	1.7 (1.1)	0.41
Surgical time (min)	184.3 (97.6)	160.7 (79.8)	0.3	184.3 (97.6)	185.5 (94.6)	0.01
Anesthetic parameter						
Hypotension *	154 (20.1%)	938 (31.3%)	0.24	154 (20.1%)	149 (19.5%)	0.016
Hypothermia ^†^	253 (33%)	1140 (38%)	0.1	253 (33%)	254 (33.2%)	0.002
Transfusion (mL)	88.4 (451)	46.9 (210)	0.2	88.4 (451)	88.8 (316)	<0.001
Crystalloid (mL)	1498 (790.2)	1167.7 (652.8)	0.5	1498 (790.2)	1498 (850)	<0.001
Colloid (mL)	242.4 (394.8)	248 (354.1)	0.02	242.4 (394.8)	271.2 (380.8)	0.07
Blood loss (mL)	339.7 (598.9)	341 (422)	0.003	339.7 (598.9)	366.1 (468.7)	0.05
Ephedrine	628 (82%)	2379 (79%)	0.07	628 (82%)	639 (83.4%)	0.038
Phenylephrine	470 (61.4%)	1649 (55%)	0.13	470 (61.4%)	472 (61.6%)	0.005
Norepinephrine	94 (12.3%)	272 (9.1%)	0.11	94 (12.3%)	94 (12.3%)	<0.001

Values are presented as mean ± SD or number (proportion). ASD < 0.2 indicate the balance of two groups. * Mean blood pressure < 55 mmHg. ^†^ Body temperature < 35 °C. Abbreviation: PSM, propensity score matching; TIVA, total intravenous anesthesia; ASD, absolute standardized mean difference; BMI, body mass index; ASA, American society of anesthesiologists; NSAIDs, non-steroidal anti-inflammatory drugs; ACEi/ARB, angiotensin-converting enzyme inhibitor/angiotensin receptor blocker.

**Table 2:** Comparison incidence of acute kidney injury (AKI) between total intravenous anesthesia (TIVA) group and volatile anesthesia group for propensity score matched cohort.

	TIVA (*n* = 766)	Volatile (*n* = 766)	*p*-Value
Total AKI	8 (1%)	32 (4.2%)	<0.001 *
AKI stage 1	7 (0.9%)	32 (4.2%)	
AKI stage 2	1 (0.1%)	0 (0%)	

Values are presented as number (proportion). * *p*-value < 0.05. Abbreviation: TIVA, total intravenous anesthesia; AKI, acute kidney injury.

**Table 3:** Univariate and multivariate logistic regression analysis for the occurrence of postoperative acute kidney injury after spine surgery.

Variables	Univariate Model		Multivariate Model	
	Odd Ratio (95% CI)	*p*-Value	Odd Ratio (95% CI)	*p*-Value
Age over 70 years	1.27 (0.88–1.81)	0.2		
Male sex (vs. female)	1.57 (1.09–2.27)	0.02 *	1.85 (1.18–3.06)	<0.001
BMI	0.99 (0.99–1.01)	0.95		
ASA physical status				
1				
2	3.02 (1.68–5.44)	<0.001 *		
3 + 4	5.35 (2.73–10.47)	<0.001 *		
Comorbidities				
Hypertension	3.17 (2.11–4.75)	<0.001 *	2.48 (1.56–3.94)	<0.001
Diabetes mellitus	2.06 (1.43–2.96)	<0.001 *		
Coronary disease	1.54 (0.92–2.56)	0.1		
Dyslipidemia	0.83 (0.38–1.79)	0.63		
Smoking	1.56 (1.1–2.23)	0.01 *		
Anemia	2.58 (1.78–3.76)	<0.001 *	2.66 (1.76–4.04)	<0.001
Medication				
NSAIDs	0.82 (0.48–1.4)	0.47		
ACEi/ARB	1.51 (1.02–2.23)	0.04 *		
Diuretics	1.9 (1.11–3.25)	0.02 *		
Surgical parameter				
Level count	1.17 (1.01–1.35)	0.04 *		
Surgical time >3 h	1.5 (1.05–2.14)	0.025 *		
Anesthetic parameter				
Hypotension †	1.58 (1.1–2.28)	0.01 *		
Hypothermia ‡	1.09 (0.75–1.56)	0.66		
Transfusion	1.00 (0.99–1.00)	0.15		
Crystalloid	1.00 (0.99–1.00)	0.12		
Colloid	1.00 (0.99–1.00)	0.89		
Estimated blood loss	1.00 (0.99–1.00)	0.35		
Ephedrine	1.02 (0.65–1.59)	0.94		
Phenylephrine	0.94 (0.66–1.34)	0.72		
Norepinephrine	1.37 (0.8–2.34)	0.25		
Anesthetic agent: volatile (vs. TIVA)	3.88 (1.89–7.98)	<0.001 *	4.69 (2.24–9.83)	<0.001

* Covariates of *p* < 0.1 were included in final multivariable logistic regression model. † Mean blood pressure < 55 mmHg, ‡ Body temperature < 35 °C. Abbreviation: BMI, body mass index; ASA, American society of anesthesiologists; NSAIDs, non-steroidal anti-inflammatory drugs; ACEi/ARB, angiotensin-converting enzyme inhibitor/angiotensin receptor blocker; TIVA, total intravenous anesthesia.

## Data Availability

The raw data of this research will be available by the first author J.H., to any qualified researcher upon reasonable request.
